# P-1605. A Phase 1, Randomized, Double-Blind, Placebo-Controlled Study to Assess the Safety, Tolerability, Pharmacokinetics, and Immunogenicity of REGN17092, an YTE modified Anti-SARS-COV-2 (COVID-19) Monoclonal Antibody in Adult Healthy Volunteers

**DOI:** 10.1093/ofid/ofaf695.1784

**Published:** 2026-01-11

**Authors:** Flonza Isa, Jan de Hoon, Ashley Sconzo, Yogesh Patel, Maria Rosario, Kenneth C Turner, Oleg Milberg, Ana Gonzalez Ortiz, Lori Faria, Ingeborg Heirman, Alina Baum, Thomas Norton, Veronica Mas Casullo, Boaz Hirshberg

**Affiliations:** Regeneron Pharmaceuticals, Inc., Tarrytown, NY; Center for Clinical Pharmacology, University Hospitals Leuven, Leuven, Luxembourg, Belgium; Regeneron Pharmaceuticals, Inc., Tarrytown, NY; Regeneron Pharmaceuticals, Inc., Tarrytown, NY; Regeneron Pharmaceuticals, Inc., Tarrytown, NY; Regeneron Pharmaceuticals Inc., Tarrytown, New York; Regeneron Pharmaceuticals, Inc., Tarrytown, NY; Regeneron Pharmaceuticals, Inc., Tarrytown, NY; Regeneron Pharmaceuticals, Inc., Tarrytown, NY; Regeneron Pharmaceuticals, Inc., Tarrytown, NY; Regeneron Pharmaceuticals, Inc., Tarrytown, NY; Regeneron Pharmaceuticals, Inc., Tarrytown, NY; Regeneron Pharmaceuticals, Inc., Tarrytown, NY; Regeneron Pharmaceuticals, Inc., Tarrytown, NY

## Abstract

**Background:**

REGN17092 is a human IgG 1 monoclonal antibody which includes a YTE modification in the Fc portion to extend the half-life. It targets severe acute respiratory syndrome coronavirus 2 (SARS-CoV-2).

**Methods:**

This study is a randomized, double-blind, placebo-controlled, single ascending dose study to evaluate the safety, tolerability and pharmacokinetics of REGN17092 in healthy subjects (18-60 yrs of age). The study consisted of 5 study cohorts evaluating intravenous (lV) dosing (300mg, 1200mg and 2400mg) and subcutaneous (SC) dosing (300mg and 1200mg) of REGN17092. The primary end point was safety and tolerability. Secondary end points included pharmacokinetics and anti-drug antibodies.

**Results:**

Between Nov 2023 and Feb 2024, a total of 78 participants screened; 40 subjects were randomized. Clinical adverse events were reported by 34/40 (85%) subjects. Most frequently reported adverse events, assessed as related by the investigator, in ≥3 participants included injection site reactions (ISR) (17.5%) and headache (10%).). Most clinical adverse events were mild to moderate in intensity. Infusion related reactions (all mild) occurred in 9 subjects (4 headache, 2 diastolic hypotension, 1 blood pressure increase, 1 dysgeusia, 1 migraine). Injection site reactions (all mild) occurred in 13 subjects. Most frequent injection site reaction symptoms reported include erythema, edema and pain. There were 2 serious adverse events (atrial fibrillation and cholecystectomy), neither were deemed related to investigational product. The REGN17092 exhibited an extended half-life of greater than 70 days.

**Conclusion:**

REGN17092 is well tolerated in healthy adults. The pharmacokinetic analyses suggest the REGN17092 maintains high plasma concentrations for several months after a single subcutaneous administration.

**Disclosures:**

Flonza Isa, MD, Regeneron Pharmaceuticals, Inc.: Stocks/Bonds (Public Company) Ashley Sconzo, MS, Regeneron Pharmaceuticals, Inc.: Stocks/Bonds (Public Company) Yogesh Patel, PhD, Regeneron Pharmaceuticals, Inc.: Stocks/Bonds (Public Company) Maria Rosario, PhD, Regeneron Pharmaceuticals, Inc.: Stocks/Bonds (Public Company) Kenneth C. Turner, PhD, Regeneron Pharmaceuticals, Inc.: Stocks/Bonds (Public Company) Oleg Milberg, PhD, Regeneron Pharmaceuticals, Inc.: Stocks/Bonds (Public Company) Ana Gonzalez Ortiz, MD, PhD, Regeneron Pharmaceuticals, Inc.: Stocks/Bonds (Public Company) Lori Faria, BS, Regeneron Pharmaceuticals, Inc.: Stocks/Bonds (Public Company) Ingeborg Heirman, PhD, Regeneron Pharmaceuticals, Inc.: Stocks/Bonds (Public Company) Alina Baum, PhD, Regeneron Pharmaceuticals, Inc.: Stocks/Bonds (Public Company) Thomas Norton, MD, Regeneron Pharmaceuticals, Inc.: Stocks/Bonds (Public Company) Veronica Mas Casullo, MD, Regeneron Pharmaceuticals, Inc.: Stocks/Bonds (Public Company) Boaz Hirshberg, MD, MBA, Regeneron Pharmaceuticals, Inc.: Stocks/Bonds (Public Company)

